# Identification and characterization of *Daldinia eschscholtzii* isolated from skin scrapings, nails, and blood

**DOI:** 10.7717/peerj.2637

**Published:** 2016-12-20

**Authors:** Kee Peng Ng, Chai Ling Chan, Su Mei Yew, Siok Koon Yeo, Yue Fen Toh, Hong Keat Looi, Shiang Ling Na, Kok Wei Lee, Wai-Yan Yee, Chee Sian Kuan

**Affiliations:** 1 Department of Medical Microbiology, University of Malaya, Kuala Lumpur, Malaysia; 2 School of Biosciences, Taylor’s University, Lakeside Campus, Subang Jaya, Malaysia; 3 Codon Genomics, Selangor, Malaysia

**Keywords:** *Daldinia eschscholtzii*, Internal transcribed spacer, Plylogenetic analysis

## Abstract

**Background:**

*Daldinia eschscholtzii* is a filamentous wood-inhabiting endophyte commonly found in woody plants. Here, we report the identification and characterization of nine *D. eschscholtzii* isolates from skin scrapings, nail clippings, and blood.

**Methods:**

The nine isolates were identified based on colony morphology, light microscopy, and internal transcribed spacer (ITS)-based phylogeny. *In vitro* antifungal susceptibility of the fungal isolates was evaluated by the Etest to determine the minimum inhibitory concentration (MIC).

**Results:**

The nine isolates examined were confirmed as *D. eschscholtzii*. They exhibited typical features of *Daldinia* sp. on Sabouraud Dextrose Agar, with white felty colonies and black-gray coloration on the reverse side. Septate hyphae, branching conidiophore with conidiogenous cells budding from its terminus, and nodulisporium-like conidiophores were observed under the microscope. Phylogenetic analysis revealed that the nine isolates were clustered within the *D. eschscholtzii* species complex. All the isolates exhibited low MICs against azole agents (voriconazole, posaconazole, itraconazole, and ketoconazole), as well as amphotericin B, with MIC of less than 1 µg/ml.

**Discussion:**

Early and definitive identification of *D. eschscholtzii* is vital to reducing misuse of antimicrobial agents. Detailed morphological and molecular characterization as well as antifungal profiling of *D. eschscholtzii* provide the basis for future studies on its biology, pathogenicity, and medicinal potential.

## Introduction

Members of the genus *Daldinia* are pyrenomycetes, which are characterized by internal horizontally zonated stromata that develop conspicuously on woody plants (Stadler et al., 2014). *Daldinia* spp. are initial colonizers as evident from the early appearance of stromata following stress or damage to the woody host plant. Initial colonization is a trait of *Daldinia* spp. owing to their habit as endophytes (Stadler et al., 2014). As an early colonizer, they remain dormant in the host without triggering symptoms before wood decay. Formation of stromata on the woody host plant is triggered by dehydration that may be caused by climatic stress, fire, or lightning (Johannesson, Læssøe & Stenlid, 2000; Srutka, Pazoutova & Kolarik, 2007). At this stage, *Daldinia* spp. becomes wood-decaying in its habit, and produces anamorphic structures under favorable conditions of humidity and temperature to colonize the substrate further (Stadler et al., 2014).

*D. eschscholtzii* is a wood-inhabiting endophyte or wood-decaying fungus that is widespread in warm tropical climate (Stadler et al., 2014). It is characterized by colonies that are white to smoky gray with a slight olivaceous-tone, and by conidiogenous structures with a nodulisporium-like branching pattern (Ju, Rogers & Martin, 1997; Stadler et al., 2014). *D. eschscholtzii* grows preferentially on dead or decaying wood substrates, and is commonly isolated from dead woody plants such as dicotyledonous crop plants, trees, and occasionally, marine algae (Karnchanatat et al., 2007; Tarman et al., 2012; Zhang et al., 2008).

Compelling data in the last decade has demonstrated the presence of a wide array of secondary metabolites in this fungus, such as 1,1′-binaphthalene-4,4′-5,5′-tetrol (BNT) (a polyketide derived from 1,8-dihydroxynaphthalene biosynthesis), cytochalasins (metabolites of mixed polyketide/NRPS origin), concentricols (terpenoids derived from the acetate-mevanolate pathway), dalesconol A and B (polyketides), and helicascolide C (polyketides) (Fang et al., 2012; Stadler et al., 2001a; Stadler et al., 2001b; Tarman et al., 2012; Zhang et al., 2008; Zhang et al., 2011). Some of these secondary metabolites are precursors of biologically active medicinal compounds. Dalesconol A and B have immunosuppressive activity (Zhang et al., 2008; Zhang et al., 2011) while helicascolide C exhibits antifungal activity against the phytopathogenic fungus *Cladosporium cucumerinum* (Tarman et al., 2012). In a previous study, genome analysis of *D. eschscholtzii* clinical isolates showed that our isolates UM 1400 and UM 1020 are potentially rich in secondary metabolites (Chan et al., 2015). The presence of the gene encoding lovastatin nonaketide synthase suggests that these isolates can synthesize the drug lovastatin that is used to induce a hypocholesterolemic effect (Chan et al., 2015).

*D. eschscholtzii* had not been reported as a human pathogen until we isolated this species from skin scrapings and the blood of patients with suspected fungal infections (Chan et al., 2015; Ng et al., 2012; Yew et al., 2014). To the best of our knowledge, all previous isolations of *D. eschscholtzii* from humans were by our group (Chan et al., 2015; Ng et al., 2012; Yew et al., 2014). Nevertheless, the clinical evidence of infection caused by this fungus remains unclear. In this study, we identified a total of nine *D. eschscholtzii* clinical isolates, including the aforementioned isolates in the past five years. Here, we present a detailed morphological, molecular, phenotypic characterization, and antifungal susceptibility profile of *D. eschscholtzii*. These data may serve as a reference for the mycological research community for rapid detection of *D. eschscholtzii*.

## Materials & Methods

###  Ethical statement

The isolates used in this study were obtained from an archived fungal collection. No patient information is disclosed except for specimen type. As such, this study is exempt from ethical approval by the UMMC Medical Ethics Committee.

### Fungal isolates

UM 230, UM 1020, UM 1094, UM 1104, UM 1134, UM 1216, UM 1217, UM 1218, and UM 1400 were isolated from skin scrapings, nail clippings, and blood of patients with suspected fungal infection in the Mycology diagnostic laboratory, UMMC, Kuala Lumpur, Malaysia (Table 1). The isolates were processed according to the laboratory’s standard operating procedures (SOP) (Yew et al., 2014) with direct wet mount microscopy followed by culture on Sabouraud Dextrose Agar (SDA; Oxoid Ltd., Basingstoke, UK) for incubation at 30 °C for seven days. The isolates were archived at 4 °C in SDA slants and maintained by periodic subculturing on SDA slants at 30 °C. UM 1020 and UM 230 isolates were not included in the morphological study as both isolates were no longer viable at the point of analysis.

**Table 1:** Clinical isolates of *D. eschscholtzii* isolated in this study.

Isolate	Source	Year	GenBank accession number	Reference
UM 1020^a^	Blood	2010	JX966563.1	Chan et al. (2015); Ng et al. (2012); Yew et al. (2014)
UM 230^a^	Nail clipping	2011	JX966562.1	Yew et al. (2014)
UM 1400	Skin scraping	2012	JX966561.1	Chan et al. (2015); Ng et al. (2012)
UM 1094	Skin scraping	2014	KT936494	Present study
UM 1104	Skin scraping	2015	KT936495	Present study
UM 1134	Skin scraping	2015	KT936496	Present study
UM 1216	Nail clipping	2015	KT936497	Present study
UM 1217	Nail clipping	2015	KT936498	Present study
UM 1218	Nail clipping	2015	KT936499	Present study

**Notes.**

a Inviable isolates, the morphological study and unique DNA signature evaluation for these two isolates were excluded in this study while the antifungal susceptibility Etest MIC readings were adopted from previous study (Yew et al., 2014).

### Morphological study

Morphological and colony features such as color, texture, and topography of the isolates were examined on SDA, potato dextrose agar (PDA; Difco Laboratories, Detroit, MI), and V8 juice agar (V8; HiMedia Laboratories, Mumbai, India). The isolates were incubated at 30 °C with alternate-day examination for fungal growth. Slide cultures of the fungi on SDA, PDA, and V8 agar were performed as previously described (Kuan et al., 2015). After a 7-day incubation at 30 °C, the fungal slide cultures were stained with lactophenol cotton blue stain and examined under the light microscope (Leica DM3000 Led, Germany).

### DNA extraction

DNA extraction was carried out as previously described (Yew et al., 2014). The pure cultures on SDA were harvested by scraping the mycelia from the agar surface and transferred to phosphate buffer saline (PBS, pH 7.4). The mycelial suspension was then transferred into a 15 ml centrifuge tube containing washed glass beads and then vortexed for five minutes. Subsequently, a total of 200 µl of lysate was subjected to DNA extraction using ZR Fungal/Bacterial DNA MiniPrep™ (Zymo Research, USA) according to the manufacturer’s protocol.

### PCR amplification and DNA sequencing

The ITS1-5.8S-ITS2 region was PCR amplified from the isolates’ genomic DNA in a 25 µl reaction consisting of 10× PCR buffer, 10 µM each of ITS1 (5′-TCCGTAGGTGAACCT GCGG-3′) and ITS4 (5′-TCCTCCGCTTATTGATATGC-3′) primers (White et al., 1990), 25 mM MgC1_2_, 2 mM deoxynucleoside triphosphate, 2.5 unit of HotStarTaq DNA polymerase, and 10 µg of each genomic DNA. The PCR was performed for 30 cycles at 94 °C for 30 s, 58 °C for 30 s, and 72 °C for 60 s. The PCR products were then purified using Expin™ PCR SV (GeneAll, Korea), and confirmed by Sanger sequencing (First Base Laboratories Kuala Lumpur, Malaysia). TraceTuner version 3.0.6 (Denisov, Arehart & Curtin, 2004) was used for base and quality calling of the sequenced ITS. The low-quality called bases (Phred value < 20) of both ends of the sequences were then trimmed by running Lucy version 1.20 (Chou & Holmes, 2001) and the included zapping.awk script. The processed ITS sequences were searched against the NCBI non-redundant (nr) nucleotide database using the nucleotide BLAST program.

### Phylogenetic analysis

Unique ITS nucleotide sequences from the isolates, together with an additional 72 reference sequences for the ITS region of *Daldinia* spp., were compiled for ITS-based phylogenetic analysis (Table 2). Two *Hypoxylon fragiforme* sequences were used as outgroup strains in the analysis (Table 2). Multiple sequence alignments of all ITS sequences were performed using M-Coffee (Moretti et al., 2007). The alignments were then trimmed using trimAl version 1.4.rev10 to remove the alignment regions with ≥50% gaps (Capella-Gutierrez, Silla-Martinez & Gabaldon, 2009). The trimmed alignments were subsequently used for phylogenetic analysis conducted using MrBayes version 3.2.1 (Huelsenbeck & Ronquist, 2001). Bayesian Markov chain Monte Carlo (MCMC) analysis was initiated by sampling across the entire general time reversible (GTR) model space. A total of 1,500,000 generations were run with a sampling frequency of 100, and diagnostics were calculated for every 1,000 generations. The first 2,500 trees were discarded with a burn-in setting of 25%. Convergence was assessed with a standard deviation of split frequencies below 0.01, no noticeable trend in the generation versus log probability of the data plot, and a potential scale reduction factor (PSRF) close to 1.0 for all parameters (Ronquist, Huelsenbeck & Teslenko, 2011).

**Table 2:** Details of isolates subjected to ITS-based phylogenetic analysis.

Fungal species	^e^Isolate	GenBank accession no.	References
*Daldinia albofibrosa*	CBS117737	JX658518.1	Stadler et al. (2014)
*Daldinia albofibrosa*	MUCL:43509 (T)	JX658451.1	Stadler et al. (2014)
^a^*Daldinia andina*	CBS114736	AM749918.1	Bitzer et al. (2008)
*Daldinia asphalatum*	MUCL:47964	JX658544.1	Stadler et al. (2014)
*Daldinia asphalatum*	MUCL:47966	JX658548.1	Stadler et al. (2014)
*Daldinia australis*	ICMP 18263 (PT)	JX658541.1	Stadler et al. (2014)
*Daldinia australis*	CBS119013 (T)	JX658450.1	Stadler et al. (2014)
*Daldinia bambusicola*	CBS 122872 (T)	JX658436.1	Stadler et al. (2014)
*Daldinia barkalovii*	CBS116999 (T)	JX658537.1	Stadler et al. (2014)
*Daldinia caldariorum*	ATCC 36660	AM749933.1	Bitzer et al. (2008)
*Daldinia caldariorum*	CBS122874	JX658452.1	Stadler et al. (2014)
*Daldinia carpinicola*	CBS122880 (T)	JX658442.1	Stadler et al. (2014)
*Daldinia* cf. *australis*	MUCL:53761	JX658547.1	Stadler et al. (2014)
*Daldinia* cf. *caldariorum*	CBS113045	JX658453.1	Stadler et al. (2014)
*Daldinia* cf. *concentrica*	MUCL:45434	JX658473.1	Stadler et al. (2014)
*Daldinia* cf. *dennisii* var. *microspora*	ICMP18265	JX658539.1	Stadler et al. (2014)
*Daldinia* cf. *eschscholtzii*	KC1690	JX658456.1	Stadler et al. (2014)
*Daldinia* cf. *grandis*	IMCP18266	JX658543.1	Stadler et al. (2014)
*Daldinia* cf. *mexicana*	Ww3844/MUCL	JX658460.1	Stadler et al. (2014)
*Daldinia* cf. *pyrenaica*	MUCL:47221	JX658515.1	Stadler et al. (2014)
*Daldinia* cf. *pyrenaica*	MUCL:51700	JX658516.1	Stadler et al. (2014)
*Daldinia childiae*	CBS116725	AM749932.1	Bitzer et al. (2008)
*Daldinia childiae*	MUCL:48616	JX658464.1	Stadler et al. (2014)
*Daldinia clavata*	MUCL:47436	JX658546.1	Stadler et al. (2014)
*Daldinia concentrica*	CBS113277	AY616683.1	Triebel et al. (2005)
*Daldinia concentrica*	MUCL:54179	JX658471.1	Stadler et al. (2014)
*Daldinia decipiens*	MUCL:44610, CBS113046	JX658476.1	Stadler et al. (2014)
*Daldinia decipiens*	CBS122879 (PT)	JX658441.1	Stadler et al. (2014)
*Daldinia dennisii*	CBS114741 (T)	JX658477.1	Stadler et al. (2014)
*Daldinia dennisii*	CBS114742 (PT)	JX658479.1	Stadler et al. (2014)
*Daldinia dennisii* var. *microspora*	MUCL:45010	JX658478.1	Stadler et al. (2014)
*Daldinia dennisii* var. *microspora*	ICMP18264	JX658538.1	Stadler et al. (2014)
*Daldinia eschscholtzii*	Not available	AB284189.1	Karnchanatat et al. (2007)
*Daldinia eschscholtzii*	CALP11206 (ET)	HE590883.1	Stadler et al. (2014)
*Daldinia eschscholtzii*	CBS113047	AY616684.1	Triebel et al. (2005)
*Daldinia eschscholtzii*	MUCL:45434	JX658484.1	Stadler et al. (2014)
*Daldinia eschscholtzii*	CBS113042	JX658497.1	Stadler et al. (2014)
*Daldinia eschscholtzii*	CBS116032	JX658500.1	Stadler et al. (2014)
*Daldinia eschscholtzii*	MUCL:38740	JX658493.1	Stadler et al. (2014)
*Daldinia eschscholtzii*	MUCL:47965	JX658482.1	Stadler et al. (2014)
*Daldinia gelatinoides*	MUCL 46173	GQ355621.1	Stadler et al. (2014)
*Daldinia gelatinosa*	UAMH 7406	JX658458.1	Stadler et al. (2014)
*Daldinia gelatinosa*	CBS116730	JX658503.1	Stadler et al. (2014)
*Daldinia govorovae*	CBS122883 (T)	JX658443.1	Stadler et al. (2014)
*Daldinia hausknechtii*	CBS119995 (T)	JX658521.1	Stadler et al. (2014)
*Daldinia lloydii*	CBS113483	JX658457.1	Stadler et al. (2014)
*Daldinia loculata*	BJ Coppins 10274 (C), CBS 114738 (ET)	AF176965.1	Johannesson, Læssøe & Stenlid (2000)
*Daldinia loculata*	TL 4613 (C)	AF176964.1	Johannesson, Læssøe & Stenlid (2000)
^b^*Daldinia loculatoides*	BJ Coppins 8630 (E), CBS113279 (T)	AF176982.1	Johannesson, Læssøe & Stenlid (2000)
*Daldinia loculatoides*	PRM885050, CBS116729	AM407726.1	S Pazoutova, 2006, unpublished data
*Daldinia macaronesica*	Ww4196(M) (T)	JX658506	Stadler et al. (2014)
*Daldinia macaronesica*	CBS 113040 (PT)	JX658504.1	Stadler et al. (2014)
*Daldinia martinii*	CBS113041 (T)	JX658507.1	Stadler et al. (2014)
*Daldinia mexicana*	Ww3843/MUCL (T)	JX658508.1	Stadler et al. (2014)
^c^*Daldinia nemorosa*	UAMH 11227	HM114296.1	ML Davey, 2010, unpublished data
*Daldinia novae-zelandiae*	CBS 114739 (PT)	JX658509.1	Stadler et al. (2014)
*Daldinia novae-zelandiae*	CBS 122873	JX658437.1	Stadler et al. (2014)
*Daldinia palmensis*	CBS113039 (T)	JX658510.1	Stadler et al. (2014)
*Daldinia petriniae*	MUCL:49214, CBS119988	JX658512.1	Stadler et al. (2014)
*Daldinia petriniae*	MUCL:51850	JX658513.1	Stadler et al. (2014)
*Daldinia pyrenaica*	MUCL:43749 (T)	AM749927.1	Bitzer et al. (2008)
*Daldinia raimundi*	CBS 113038 (T)	JX658517.1	Stadler et al. (2014)
*Daldinia raimundi*	MUCL:51689	JX658446.1	Stadler et al. (2014)
*Daldinia starbaeckii*	MUCL:45436 (T)	JX658488.1	Stadler et al. (2014)
*Daldinia starbaeckii*	CBS116727	JX658489.1	Stadler et al. (2014)
*Daldinia steglichii*	MUCL:43512 (PT)	JX658534.1	Stadler et al. (2014)
*Daldinia steglichii*	MUCL:53886	JX658545.1	Stadler et al. (2014)
*Daldinia theissenii*	BCRC34045, CBS122875	JX658468.1	Stadler et al. (2014)
^d^*Daldinia theissenii*	CBS113044	AM749931.1	Bitzer et al. (2008)
*Daldinia vanderguchtiae*	CBS113036 (T)	JX658520.1	Stadler et al. (2014)
*Daldinia vernicosa*	CBS 161.31 (T)	JX658519.1	Stadler et al. (2014)
*Daldinia vernicosa*	CBS119316	AM749925.1	Bitzer et al. (2008)
*Hypoxylon fragiforme*	CBS114745	AY616690.1	Stadler et al. (2014)
*Hypoxylon fragiforme*	YMJ 383	JN979420.1	Hsieh & Rogers (2005)

**Notes.**

a Previously idetifies as *D. grandis* by Bitzer et al. (2008) and reclassified by Stadler et al. (2014).

b Previously identified as *D. grandis* by Johannesson, Læssøe & Stenlid (2000) and reclassified by Stadler et al. (2014).

c Previously identified as *Annelosporium nemorosum* and reclassified by Stadler et al. (2014).

d Previously identified as *D. clavata* by Bitzer et al. (2008) and reclassified by Stadler et al. (2014).

e “T” indicates type strains, “ET” indicates eipitypes and “PT” indicates paratype.

### *In vitro* antifungal susceptibility test

The Etest (bioMérieux, France) was performed according to the manufacturer’s instructions to determine the MICs of anidulafungin (ANID), amphotericin B (AMB), caspofungin (CAS), fluconazole (FLC), itraconazole (ITC), ketoconazole (KTC), posaconazole (PSC), and voriconazole (VRC). The concentration gradient of ANID, AMB, CAS, ITC, KTC, PSC, and VRC ranged from 0.002 to 32 µg/ml, while that of FLC ranged from 0.016 to 256 µg/ml. The test was performed on RPMI 1640 medium containing 2% glucose and MOPS. Each culture growing on SDA was harvested, suspended in sterile saline solution, and adjusted to a turbidity of a 0.5 McFarland standard. A sterile cotton swab was used to spread 500 µl fungal suspension evenly on a RPMI plate. Etest strips were placed on plates that had been dried for at least 10 min at room temperature. The MICs were determined after 72 h of incubation at 30 °C. Both on-scale and off-scale MICs were included in the data. For the calculation of geometric mean (GM), the high off-scale MICs (>32 and >256 µg/ml) were rounded up to the next intermediate Etest dilution (48 and 384 µg/ml), while the low off-scale MICs (<0.002 and <0.016 µg/ml) remained unchanged.

### Nucleotide sequence accession numbers

The ITS nucleotide sequences of UM 1094, UM 1104, UM 1134, UM 1216, UM 1217, and UM 1218 were deposited in the GenBank database with the accession numbers KT936494, KT936495, KT936496, KT936497, KT936498, and KT936499, respectively (Table 1).

## Results

### Morphological study

**Figure 1:**
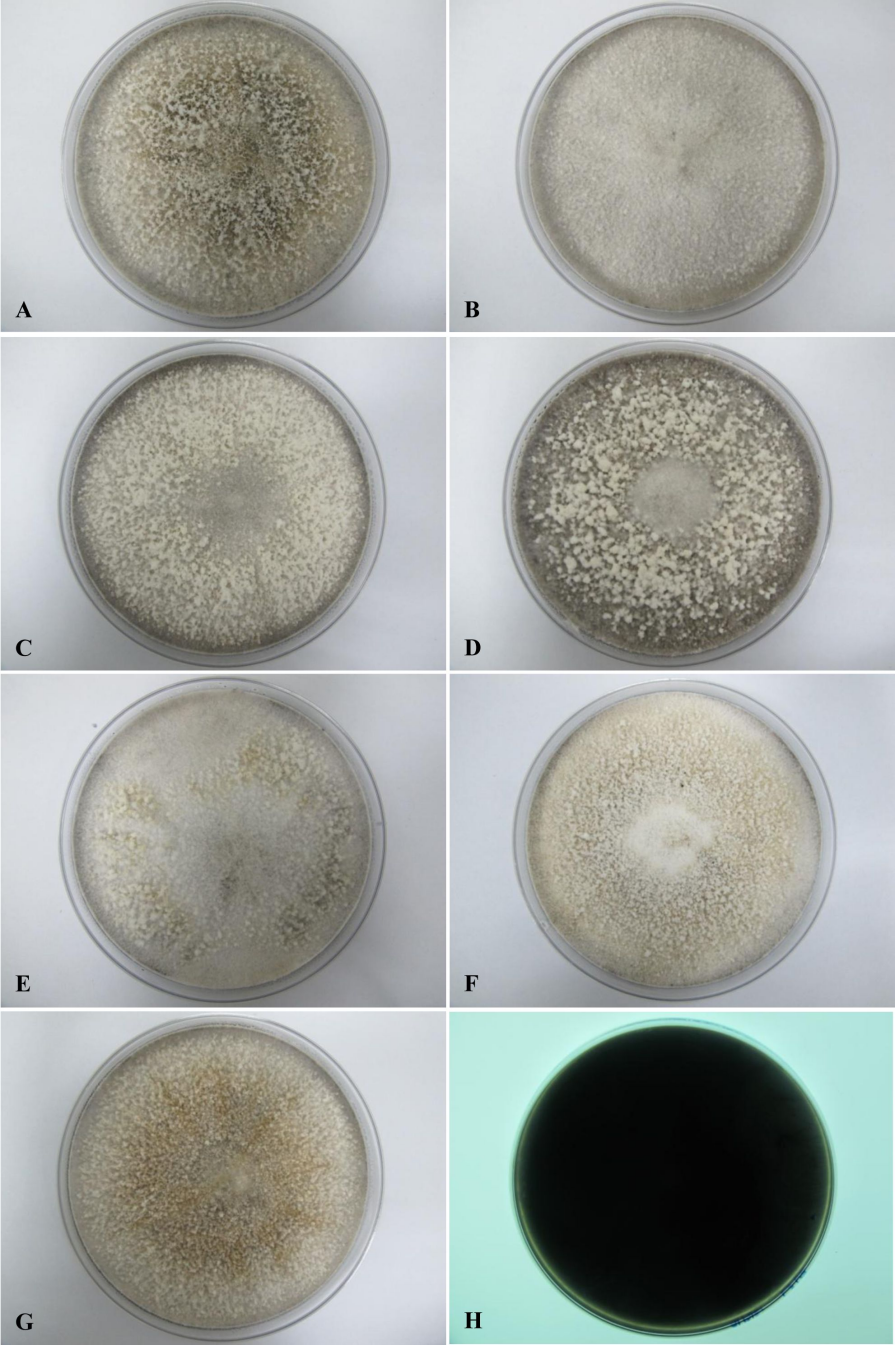
Colonial morphology of *D. eschscholtzii* isolates on SDA. (A) UM 1400, (B) UM 1094, (C) UM 1104, (D) UM 1134, (E) UM 1216, (F) UM 1217, and (G) UM 1218 were incubated at 30 °C for 5 days. (H) Black coloration on the reverse.

**Figure 2:**
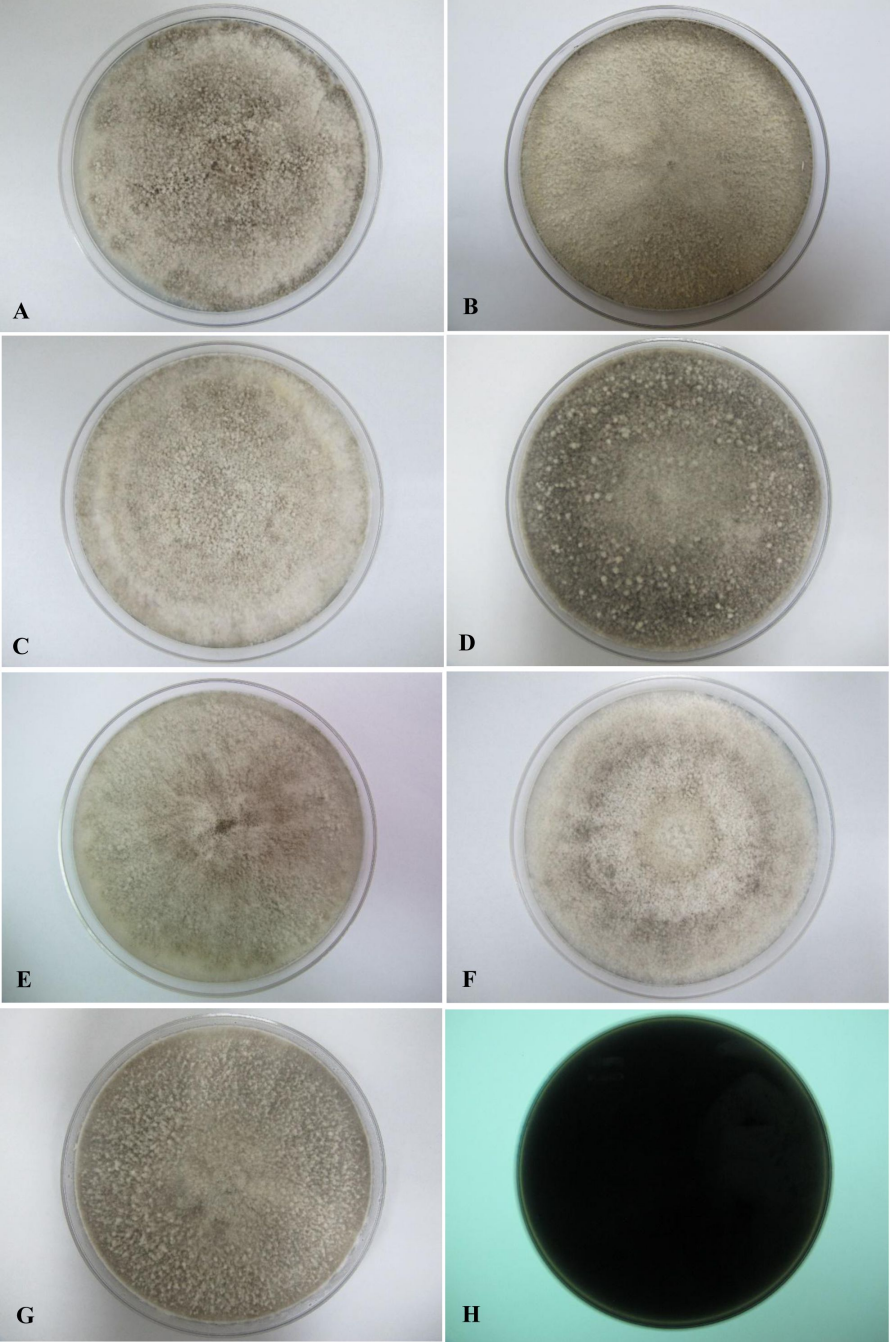
Colonial morphology of *D. eschscholtzii* isolates on PDA. (A) UM 1400, (B) UM 1094, (C) UM 1104, and (G) UM 1218 were incubated at 30 °C for 7 days. (D) UM 1134, (E) UM 1216, and (F) UM 1217 were incubated at 30 °C for 5 days. (H) Black coloration on the reverse.

**Figure 3:**
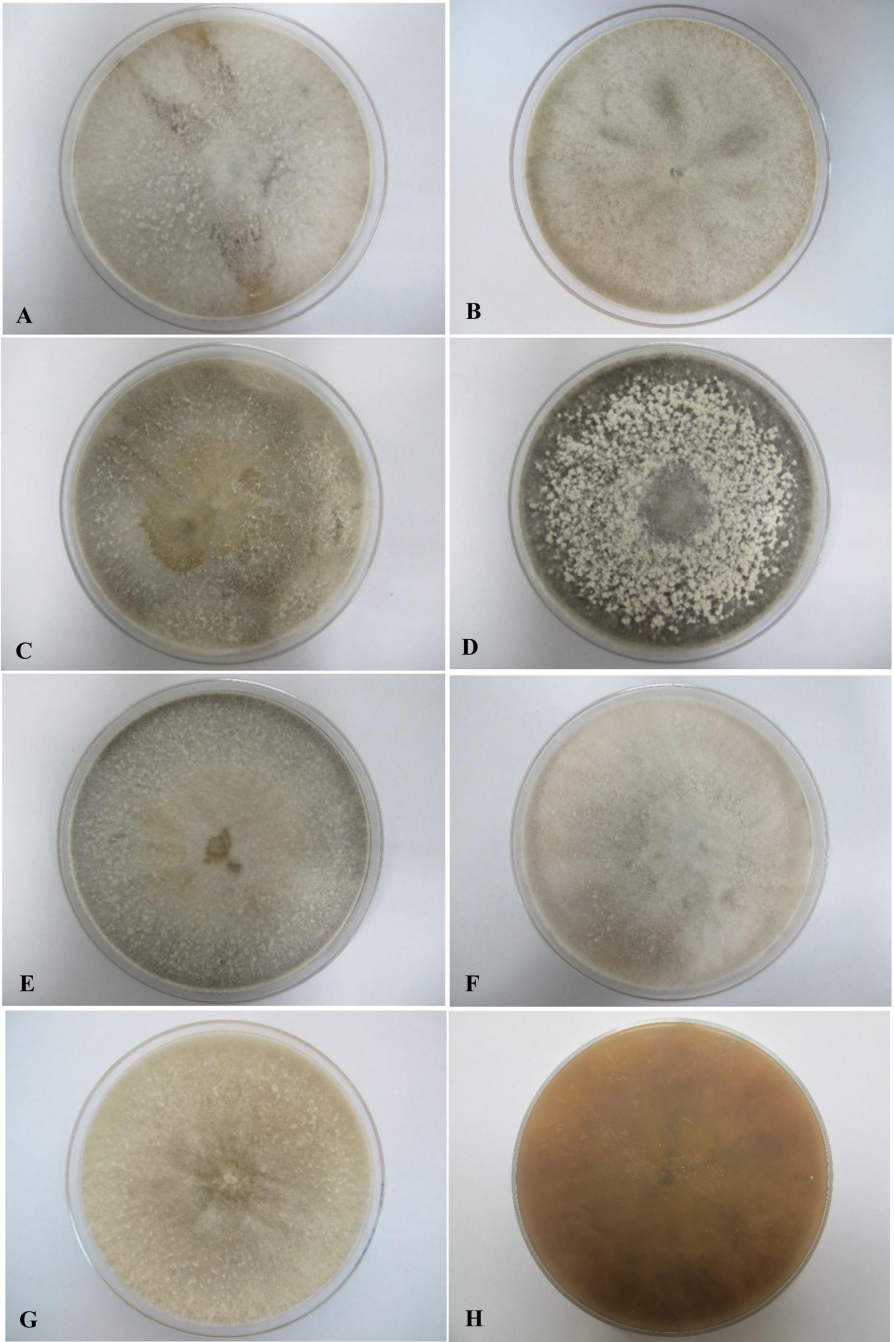
Colonial morphology of *D. eschscholtzii* isolates on V8 agar. (A) UM 1400, (B) UM 1094, (C) UM 1104, (D) UM 1134, (E) UM 1216, (F) UM 1217, and (G) UM 1218 were incubated at 30 °C for 5 days. (H) Uncolored with slight blackish on the reverse.

All seven isolates grew rapidly on SDA. Initially, white hyphae grew from the inoculated site and formed a felty azonate mycelium. With aging, it turned to smoky gray color with a slight olivaceous tone as the mycelium became fully differentiated (Fig. 1). The smoky gray coloration was indicative of sporulation. All isolates had a black-gray coloration on the reverse side. The cultural characteristics of all fungal isolates that grew on SDA were similar to those that growing on PDA plates. However, their growth rates on PDA were different. The strains UM 1134, UM 1216, and UM 1218 grew faster (five days to reach the periphery of the 9 cm plate) than UM 1400, UM 1094, UM 1104, and UM 1217 (seven days) (Fig. 2). On V8 agar, all the colonies reached the edge of plate after a 5-day incubation; they had a felty to fluffy texture appearance (Fig. 3). The surfaces of the colony changed from white to gray or black after 5 days of incubation. The reverse side of V8 agar plate was initially colorless, and became slightly black after culture for five days. UM 1134 showed denser mycelial growth on SDA, PDA, and V8 agar as compared to the other isolates (Figs. 1D, 2D and 3D).

**Figure 4:**
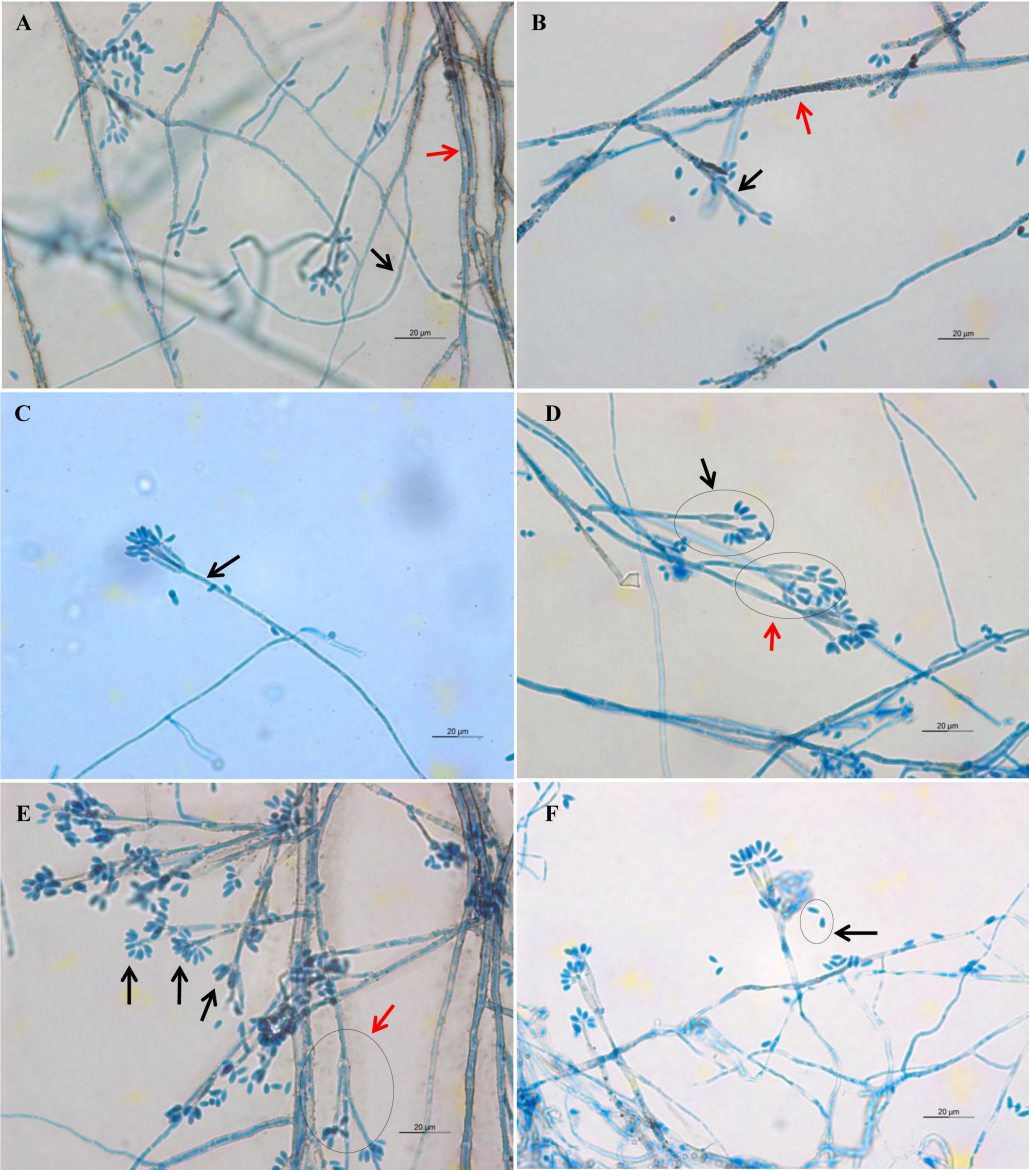
Microscopic morphology of *D. eschscholtzii* isolates. (A) Thin-walled and hyaline septate hyphae (black arrow), thick-walled and melanized septate hyphae (red arrow). (B) Pigmented exudates on hyphae surface (red arrow), additional branch grew from the conidiogenous regions (black arrow). (C) Mononematous conidiophore (black arrow) with conidiogenous cells arising from its terminus. (D) Conidiophore with dichotomous branching, with two (black arrow) to three (red arrow) conidiogenous cells arising from each terminus. (E) Conidia were produced holoblastically in sympodial sequence on the terminus of the conidiogenous cells (black arrows), conidiophore with trichotomous branching pattern (red arrow). (E) Ellipsoid conidia with attenuated base (black arrow). (400× magnification, bars 20 µm).

Light microscope analysis revealed that all isolates that grew on SDA, PDA, and V8 agar had similar morphology. The septate hyphae could be hyaline thin-walled or melanized thick-walled (Fig. 4A). The thick-walled hyphae showed black exudates on their surfaces (Fig. 4B). Septate conidiophores were irregularly branched into mononematous, dichotomous or trichotomous structures with one to three conidiogenous cells originating from the terminus (Figs. 4C–4E). The conidiophores were hyaline and black with occasional pigmented exudates. The conidiogenous cells were hyaline and cylindrical. On the apex of conidiogenous cells, conidia were produced holoblastically in a sympodial sequence (Fig. 4E). The conidia were hyaline and ellipsoid with an attenuated base as shown in Fig. 4F. Table 3 summarizes the morphological features of this fungal species.

**Table 3:** Key morphological features of clinically isolated *D. eschscholtzii*.

Culture medium	Macroscopic features	Microscopic features
Sabouraud dextrose agar	Colonies attain a diameter of 9 cm agar plate in 5 days of incubation at 30 °C. Colonies are felty and azonate. At first colonies are whitish, turning smoke gray with slight olivaceous tone in age. Reverse appears black in color.	Hyphae are septate, thin-walled and hyaline to thick-walled and melanized, with thick-walled hyphae often have blackish exudates. Conidiophores are septate, hyaline to melanized, some with blackish exudates, mononematously, dichotomously or trichotomously irregular branched, occasionally branched from conidiogenous region, bearing one to three conidiogenous cells on its terminus, up to 167 µm length × 2.2–3.3 µm diameter. Conidiogenous cells are cylindrical and hyaline, bearing conidia on its apical region, 8.9–27.8 µm length × 1.1–2.2 µm diameter. Conidia are ellipsoid, aseptate, solitary, hyaline, with attenuated base, produced holoblastically in sympodial sequence, 4.4–6.7 µm length × 1.7–2.2 µm diameter.
Potato dextrose agar	Colonies attain a diameter of 9 cm agar plate in 5–7 days of incubation at 30 °C. Colonies are felty, azonate or zonate. At first colonies are whitish, turning smoke gray with slight olivaceous tone with age. Reverse appears black in color.	
V8 juice agar	Colonies attain a diameter of 9 cm agar plate in 5 days of incubation at 30 °C. Colonies are felty to fluffy, azonate. At first colonies are whitish, later turning gray or black, some with slight olivaceous tone. Reverse is initially uncolored, later becoming slight blackish.	

**Figure 5:**
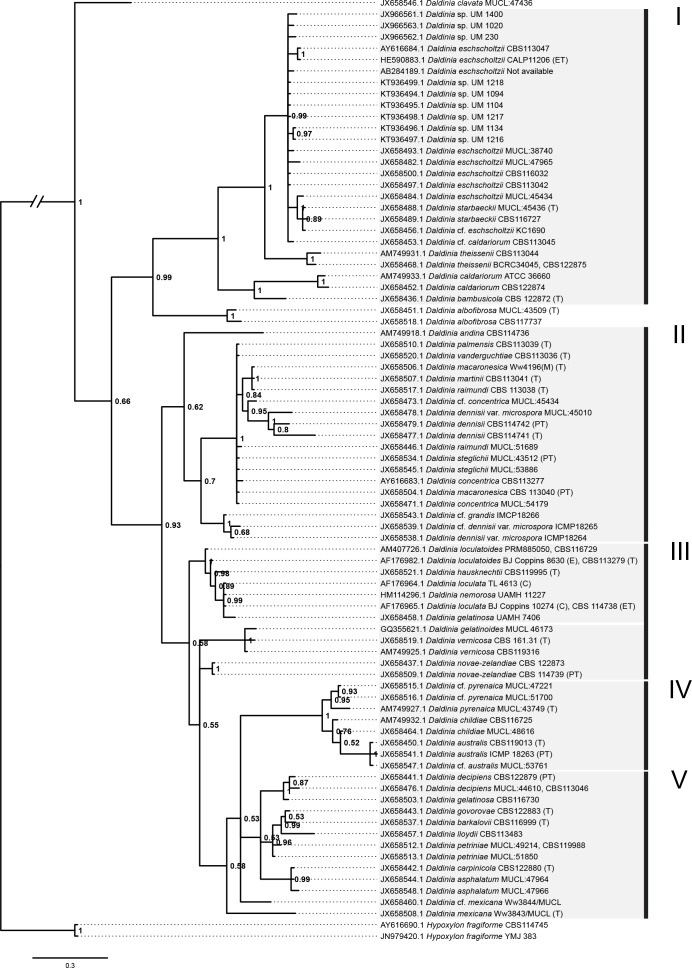
Bayesian phylogram generated using the ITS sequence data. The tree was rooted with two *Hypoxylon fragiforme* sequences as outgroups. UM 1400, UM 2010, UM 230, UM 1094, UM 1104, UM 1134, UM 1216, UM 1217, and UM 1218 isolates were clustered in Group I. Numbers on the nodes indicate Bayesian posterior probability based on 100 sampling frequency for a total of 15,000,000 generations.

### Molecular study

All isolates were identified as *D. eschscholtzii* following the BLASTn searches. The ITS-based phylogenetic tree in this study comprised members from the genus *Daldinia* (Fig. 5), and was divided into groups as described by Stadler et al. (2014), namely the *D. eschscholtzii* group (Group I), *D. concentrica* group (Group II), *D. vernicosa/loculata* group (Group III), *D. childiae* group (Group IV), and *D. petriniae* group (Group V). In this study, all the isolates were clustered within the *D. eschscholtzii* group, forming a cluster with the reference isolates of *D. eschscholtzii*.

### *In vitro* antifungal susceptibility test

The MICs of the nine isolates tested are shown in Table 4. The antifungal susceptibility profiles obtained were isolate-dependent. In general, VRC, PSC, ITC, KTC, and AMB displayed very low MICs to *D. eschscholtzii*, with all the isolates exhibiting MICs of ≤1 µg/ml. ANID also showed low MICs to (≤1 µg/ml) the different isolates, with the exception of UM 1134 and UM 1218 (>32 µg/ml). Similarly, low MIC values (≤1 µg/ml) were obtained for CAS, with two thirds of the isolates eliciting MICs of ≤1 µg/ml, and the remaining eliciting MICs of >1 µg/ml. Overall, PSC exhibited the highest *in vitro* anti-fungal activity (GM MIC 0.016 µg/ml) against all isolates, followed by VRC (GM MIC 0.021 µg/ml), KTC (GM MIC 0.027 µg/ml), AMB (GM MIC 0.031 µg/ml), ANID (GM MIC 0.062 µg/ml), ITC (GM MIC 0.089 µg/ml), and CAS (GM MIC 0.481 µg/ml). Relatively high MICs against *D. eschscholtzii* (88.89% with MIC >1 µg/ml; GM MIC value of 3.530 µg/ml) were found for FLC, indicating potential resistance of *D. eschscholtzii* to this drug.

**Table 4:** Minimum inhibitory concentration (MICs) for the isolates determined by Etest.

Antifungal agent^a^	Etest MIC (µg/ml)									GM^c^ (µg/ml)	MIC category^d^
	UM 1020^b^	UM 230^b^	UM 1400^b^	UM 1094	UM 1104	UM 1134	UM 1216	UM 1217	UM 1218		
FLC	<0.016	6	1.5	4	12	1.5	6	>256	4	3.530	B
VRC	<0.002	0.125	<0.002	0.016	0.064	0.023	0.012	0.125	0.032	0.021	A
PSC	0.004	0.064	<0.002	0.032	0.032	0.008	0.016	0.047	0.016	0.016	A
ITC	<0.002	0.5	0.064	0.125	0.125	0.023	0.125	0.38	0.25	0.089	A
KTC	0.003	0.064	0.012	0.032	0.064	0.023	0.064	0.023	0.032	0.027	A
ANID	<0.002	0.094	<0.002	<0.002	<0.002	>32	0.032	0.094	>32	0.062	A
AMB	0.125	0.19	<0.002	0.064	0.047	<0.002	0.125	0.25	<0.002	0.031	A
CAS	3	2	0.008	0.25	0.5	0.5	1.5	0.38	0.75	0.481	A

**Notes.**

a FLC, fluconazole; VRC, voriconazole; PSC, posaconazole; ITC, itraconazole; KTC, ketoconazole; ANID, anidulafungin; AMB, amphotericin B; CAS, caspofungin.

b Etest MIC values were retrieved from previous study (Yew et al., 2014).

c GM, geometric mean.

d MIC categories: Category A: ≤1 µg/ml, Category B: >1–32 µg/ml or >1–256 µg/ml, Category C: >32 µg/ml or >256 µg/ml.

## Discussion

*Daldinia eschscholtzii* is a filamentous fungus commonly found as an endophyte or a wood-decaying fungus in woody plants (Karnchanatat et al., 2007; Karnchanatat et al., 2008; Stadler et al., 2014). Although we previously reported isolations of this organism from humans, it is unclear whether it is the cause of an actual infection, or if it merely exists as a harmless colonizer living in the nail plate or skin surface damaged by trauma or other diseases. In this study, we obtained nine *D. eschscholtzii* isolates from blood specimens, skin scrapings, and nail clippings. While the clinical significance of *D. eschscholtzii* remains in question, repeated isolation of this fungal species from humans recently suggests that it is not a mere environmental contaminant in patients. Chan et al. (2015) report that the genomes of *D. eschscholtzii* harbor several stress adaptation mechanisms for their survival in human hosts. Hence, it would not be surprising for the species to have undergone rapid evolution to select for fitness attributes as well as virulence factors related to pathogenicity in humans.

Filamentous fungi are routinely identified by colony morphology and microscopy. The former would not precisely identify *D. eschscholtzii* owing to their natural variation among the isolates and tendency towards media-dependency, as evident from their macroscopic appearances. Identification to species level based on morphological examination alone would be difficult as many species of *Daldinia* are morphologically very similar (Ju, Rogers & Martin, 1997; Stadler et al., 2014), and hence considerable expertise and experience are required of the examiner in this regard.

The ITS region of the nuclear rDNA can be used to examine species level relationship in fungi due to its higher degree of variation. Thus, PCR-based ITS sequence analysis has been widely used to identify *D. eschscholtzii* (Chan et al., 2015; Hu et al., 2014; Tarman et al., 2012; Yuyama et al., 2013). Despite recent studies reporting limitations of the ITS region in distinguishing between the species complexes of *Daldinia* spp., this region has the broadest taxa covered in *Daldinia* (Stadler et al., 2014). The phylogenetic analysis showed that our clinical isolates and reference environmental isolates of *D. eschscholtzii* formed a cluster. However, further studies based on protein coding genes are needed to segregate members of the *D. eschscholtzii* species complex reliably.

The Etest is a simple, reliable, and reproducible assay that has been shown to correlate with the Clinical and Laboratory Standards Institute (CLSI) method in antifungal susceptibility testing of filamentous fungi (Espinel-Ingroff, 2001; Szekely, Johnson & Warnock, 1999). In line with this, we applied the Etest to study the antifungal profiles of our isolates. The results showed that *D. eschscholtzii* elicited low MICs in all the antifungal agents tested, except for FLC. Among these antifungals, VRC, PSC, ITC, KTC, and AMB were the more active *in vitro*, with all isolates inhibited by concentrations of less than 1 µg/ml. Since there is no available information on antifungal susceptibility profiles for *D. eschscholtzii*, this work will contribute towards establishing an optimal antifungal precautionary treatment for this fungus.

## Conclusions

In this paper, we report the isolation of *D. eschscholtzii* from superficial sites in humans, predominantly skin and nails. If these fungi are confirmed to be of clinical importance, the *in vitro* antifungal activities determined here might be useful in clinical practice. The characterization of these fungi is important to understand the basic fungal biology of *D. eschscholtzii*, and to provide clues on how they are evolutionarily adapted to the human host. The data in this study will serve as a foundation for future research on pathogenicity of *D. eschscholtzii* in humans.

## Additional Information and Declarations

### DNA Deposition

The following information was supplied regarding the deposition of DNA sequences: Six ITS sequences that were newly generated in the present study have been deposited in the NCBI GenBank database under accession numbers KT936494, KT936495, KT936496, KT936497, KT936498 and KT936499.
